# Molecular Characterization and Subtyping of Breast Cancer Cell Lines Provide Novel Insights into Cancer Relevant Genes

**DOI:** 10.3390/cells13040301

**Published:** 2024-02-06

**Authors:** Claudia Pommerenke, Stefan Nagel, Josephine Haake, Anne Leena Koelz, Matthias Christgen, Laura Steenpass, Sonja Eberth

**Affiliations:** 1 Department of Bioinformatics, IT and Databases, Leibniz Institute DSMZ-German Collection of Microorganisms and Cell Cultures, 38124 Braunschweig, Germany; claudia.pommerenke@dsmz.de; 2 Department of Human and Animal Cell Lines, Leibniz Institute DSMZ-German Collection of Microorganisms and Cell Cultures, 38124 Braunschweig, Germany; stefan.nagel@dsmz.de (S.N.);; 3 Institute of Pathology, Hannover Medical School, 30625 Hannover, Germany; 4 Zoological Institute, Technische Universität Braunschweig, 38106 Braunschweig, Germany

**Keywords:** AIM1, FGFR2-CRYBG1, homeobox, mammary carcinoma, miR-99a, PAM50, RTN4IP1-CRYBG1

## Abstract

Continuous cell lines are important and commonly used in vitro models in breast cancer (BC) research. Selection of the appropriate model cell line is crucial and requires consideration of their molecular characteristics. To characterize BC cell line models in depth, we profiled a panel of 29 authenticated and publicly available BC cell lines by mRNA-sequencing, mutation analysis, and immunoblotting. Gene expression profiles separated BC cell lines in two major clusters that represent basal-like (mainly triple-negative BC) and luminal BC subtypes, respectively. HER2-positive cell lines were located within the luminal cluster. Mutation calling highlighted the frequent aberration of *TP53* and *BRCA2* in BC cell lines, which, therefore, share relevant characteristics with primary BC. Furthermore, we showed that the data can be used to find novel, potential oncogenic fusion transcripts, e.g., *FGFR2::CRYBG1* and *RTN4IP1::CRYBG1* in cell line MFM-223, and to elucidate the regulatory circuit of *IRX* genes and *KLF15* as novel candidate tumor suppressor genes in BC. Our data indicated that *KLF15* was activated by IRX1 and inhibited by IRX3. Moreover, KLF15 inhibited *IRX1* in cell line HCC-1599. Each BC cell line carries unique molecular features. Therefore, the molecular characteristics of BC cell lines described here might serve as a valuable resource to improve the selection of appropriate models for BC research.

## 1. Introduction

Breast cancer (BC) is the most common cancer worldwide with still increasing incidences [1]. In 2020 over 2.3 million new BC cases in both sexes and 685,000 deaths from BC in women were documented globally [2]. Early-stage BC is curable in 70–80% of patients, in contrast to advanced metastatic BC, which is still considered incurable [3]. Well established biomarkers with clinical relevance in respect to targeted treatment options and prognosis are the hormone receptors ER (estrogen receptor alpha, encoded by *ESR1*) and PR (progesterone receptor, encoded by *PGR*), as well as HER2 (human epidermal growth factor receptor 2, encoded by *ERBB2*) [4]. Triple-negative breast cancer (TNBC) is characterized by the absence of the aforementioned receptors and is correlated with a worse prognosis compared to receptor-positive subtypes [4].

BC represents a genetically, histologically, and clinically heterogeneous malignancy comprising various histological and molecular subtypes. Histologic subtypes include invasive BC of no specific type (NST, formerly known as ductal BC) and invasive lobular carcinoma (ILC), which develop from distinct precursor lesions known as ductal carcinoma in situ (DCIS) and lobular carcinoma in situ (LCIS) [3,5]. Initiated by Perou et al. more than 20 years ago, intrinsic molecular subtypes that are distinguished by gene expression profiles of a panel of 50 genes, termed PAM50 gene set, soon became common for molecular subtyping of BC and prediction of outcome [6,7,8]. The PAM50 intrinsic subtypes comprise Luminal A (LumA), Luminal B (LumB), HER2-enriched, basal-like, and normal-like tumors [7]. Remarkably, intrinsic subtypes partially overlap with the immunohistochemical classification, e.g., TNBC is enriched for basal-like BC and most LumA and LumB tumors are positive for ER [4].

Continuous BC cell lines are widely used and represent valuable and essential in vitro models for BC research [9]. In contrast to primary material, continuous cell lines are of unlimited supply and can easily be modified. Highlighting the relevance of BC cell line models, the ER^+^ cell line MCF-7, which was established in 1973, was an essential tool for the development of fulvestrant as a therapeutic agent for the treatment of ER^+^ metastatic BC [10,11]. Several studies reported that BC cell lines can indeed serve as suitable in vitro models, although certain limitations must be recognized: BC cell lines and primary tumors show differences in respect to tumor cell content and heterogeneity, copy number variations (CNV), mutations, and gene expression profiles [9,12,13,14]. Because of the heterogeneity of BC, the molecular characteristics and the clinical history of each BC cell line require consideration for proper selection of appropriate cell line models for each BC research question. Although data about molecular characteristics (e.g., PAM50 subtypes) are available for the commonly used BC cell lines [12,13,14], it is important to note that some inconsistencies in subtyping and the status of relevant biomarkers were observed in the literature. This could be a result of methodological differences but also from subclones of the same cell lines circulating between different laboratories as extensively investigated for subclones of MCF-7 by Ben-David et al., who highlighted the impact of the observed genetic diversity between subclones on drug responses [15]. Consequently, there is an ongoing need for in-depth molecular characterization of publicly available BC cell lines of verified authenticity. 

Recently, we characterized 100 leukemia and lymphoma cell lines (LL-100 panel) as models for hematological neoplasms by transcriptome and exome sequencing using uniform methodology [16]. Here, applying the same standards for cell line cultivation and data generation, we characterized in depth 29 authenticated and publicly available BC cell lines by mRNA-sequencing, mutation patterns, microRNA expression analysis, and immunoblotting for molecular subtyping. The results give novel insights into the molecular landscape of BC cell lines. We included the expression data into our public web tool DSMZCellDive (https://celldive.dsmz.de/) to support the appropriate model selection for the BC research community.

## 2. Materials and Methods

### 2.1. Cell Lines and Sample Preparation

Human BC cell lines were taken from the authenticated stocks of the DSMZ cell lines bank and are listed in Table 1. Cultivation conditions and media are listed in Table S1. The STR profiles are accessible via our STR profile browser on DSMZCellDive (https://celldive.dsmz.de/str/browse, date of release 13 April 2022). Total RNA was isolated from exponentially growing cultures using the miRNeasy Mini Kit (Qiagen, Hilden, Germany) including DNase digestion following the manufacturer’s instructions. Genomic DNA (gDNA) was extracted with the QIAamp DNA Blood Mini Kit (Qiagen). Protein lysates were prepared with RIPA buffer containing protease inhibitors (Roche, Penzberg, Germany).

### 2.2. Immunoblotting

For immunoblotting, protein lysates were separated on 10–15% polyacrylamide SDS gels and electro-transferred onto PVDF membranes (Biorad, Feldkirchen, Germany) using a Mini Trans-Blot Electrophoretic Transfer Cell (Biorad). Membranes were blocked in 5% non-fat dry milk in TBS/T and probed with the respective primary antibody: anti-AR (1:2000, rabbit monoclonal IgG, D6F11; Cell Signaling Technology, Danvers, MA, USA), anti-ERα (1:1000, rabbit monoclonal IgG, D8H8; Cell Signaling Technology), anti-HER2 (1:2000, rabbit monoclonal IgG, 29D8; Cell Signaling Technology), anti-PR (1:100, rabbit monoclonal IgG, D8Q2J; Cell Signaling Technology), anti-GAPDH (1:10,000, mouse monoclonal IgG1, 6C5; Abcam, Cambridge, UK), anti-IRX1 (1:500, rabbit polyclonal, Biozol, Eching, Germany, #DF3225), anti-IRX2 (1:1000, rabbit polyclonal, Biozol, #LS-C800571), anti-IRX3 (1:500, rabbit polyclonal, Biozol, #MBS8223417), anti-KLF15 (1:1000, mouse monoclonal IgG2a, A-5, Santa Cruz Biotechnology, Dallas, TX, USA), anti-TUBA (1:1000, mouse monoclonal, Sigma, St. Louis, MO, USA, #T6199). Secondary antibodies were purchased from GE Healthcare Life Sciences and used at 1:10,000 dilution (anti-mouse IgG HRP-linked NXA931, anti-rabbit IgG HRP-linked NA934). Proteins were detected by chemiluminescence (Western lightning Plus ECL Solution; Perkin Elmer, Waltham, MA, USA) in the Advanced Fluorescence Imager machine (Intas, Göttingen, Germany). Signal intensities were quantified by densitometric analysis using ImageJ v1.52a and GAPDH signals as loading control. Protein levels were expressed in relation to the levels detected in BT-474, which was set to 1. Thresholds for relative protein levels of AR, ER, and PR were then set as follows: 0.1 < (+) < 0.5 < (++) < 2 < (+++). Due to the strong expression of HER2 in BT-474, thresholds for relative protein levels of HER2 in respect to levels in BT-474 were set as follows: 0.01 < (+) < 0.2 < (++) < 0.5 < (+++). 

### 2.3. mRNA-Sequencing and Expression Analysis

Library preparation and mRNA-sequencing (RNA-seq) was performed by Eurofins Genomics. Briefly, strand-specific mRNA-libraries were prepared with the NEBNext Ultra II Directional RNA Library Prep Kit for Illumina (New England Biolabs, Frankfurt am Main, Germany), amplified and sequenced on a NovaSeq 6000 (Illumina, München Germany) with 2 × 150 cycles (paired-end) with at least 30 million reads per sample. Insert sizes were aimed at 2 × 150 bp length in order to increase the probability to capture fusion genes and to achieve non-redundant reads for variant calling [33]. Preprocessing and analysis were conducted as described previously [34] and the pipeline can be retrieved at zenodo (https://zenodo.org/records/6401600, date of release 31 March 2022). Briefly, reads were trimmed with fastq-mcf, ea-utils, checked for quality using FastQC, quantified with Salmon, and analysed with R/Bioconductor package DESeq2. RNA-seq reads are accessible at BioStudies under S-BSST1200C. Processed expression data are available via the web tool DSMZCellDive [34].

### 2.4. Quantitative PCR

For gene expression analysis cDNA was synthesized by random priming from 1 µg RNA using Superscript II (Invitrogen). For miRNA expression analysis cDNA was prepared with the miRCURY LNA RT Kit (Qiagen, Hilden, Germany) according to the manufactures’ instructions. Real-time quantitative PCR (qPCR) analysis was performed with the 7500 Real-time System (Thermo Fisher Scientific, Dreieich, Germany). For gene expression analyses Taqman gene expression assays and Taqman Fast Advanced Master Mix (Thermo Fisher Scientific) were applied using TBP as endogenous control. For miRNA expression analysis, the miRCRUY LNA SYBR Green PCR Kit and respective miRCURY Primer assays (Qiagen) were used with SNORD48 as endogenous control. Analyses were performed in triplicate and expression data were evaluated using the ddCt-method. Standard deviations were calculated for each experiment and presented in the figures as error bars. Statistical significance was assessed by t-test and the derived *p*-values are indicated by asterisks (* *p* < 0.05, ** *p* < 0.01, *** *p* < 0.001, n.s. not significant). 

### 2.5. Mutation Calling

Data obtained from RNA-seq were used to identify mutations transcribed to mRNA. Single nucleotide variants (SNVs), as well as small insertions and deletions (InDels) were identified by the HaplotypeCaller of GATK. Sequencing reads were prepared by trimming the sequences with fastp [35], mapping via STAR-aligner in two-pass mode [36], read group adding, duplicate labeling, splitting reads with N in cigar, base recalibration, using HaplotypeCaller, filtering variants following GATK best practices for RNA-seq short variant discovery [37]. RNA-edit sites and regions with <5 read depth were excluded from variant calling for quality reasons. For filtering, common variants data from 1000 genomes project phase3 [38], gnomAD r2.1.1 [39], and dbSNP v156 [40] were taken setting the allele frequency > 0.01 using SnpSift [41], snpEff [42], vcftools [43], vcf2maf [44], and VEP [45]. In addition variants occurring in more than one third of the samples were removed, since many of these variants were located in homopolymer or repetitive regions. Furthermore, if present, only variants with predicted functional consequences of SIFT ≤ 0.02 or PolyPhen ≥ 0.2 were kept. Focusing on coding regions, the mutation types “Nonsense Mutation”, “Frame Shift Ins”, “Frame Shift Del”, “In Frame Ins”, “In Frame Del”, “Translation Start Site”, “Splice Site”, “Missense Mutation” were kept. The waterfall plot was visualized with the R package GenVisR [46].

### 2.6. Fusion Calling and Analysis of Fusion Transcripts

In order to find novel somatic fusion genes in RNA-seq data, we applied FusionCatcher (v1.30) which scans the paired-end reads for fusion junctions by four different aligners: Bowtie, Bowtie2, BLAT, and STAR [47]. Additionally, FusionCatcher contains preprocessing steps such as trimming and assigns public data from Ensembl, UCSC, RefSeq and further. 

For validation, predicted fusion transcripts were amplified from cDNA and gDNA with primers designed to anneal in the respective complementary exons at either side of the predicted fusion. *ABL1* primers served as internal control for the template material. Primers are listed in Table 2. Briefly, cDNA was prepared from total RNA with Superscript II (Invitrogen) and amplified using TaKaRa Taq HS polymerase kit (Takara, Saint-Germain-en-Laye, France) in a C1000 (Bio-Rad) with 40 cycles and an annealing temperature of 60.7 °C for the fusion genes and 70 °C for *ABL1*, respectively. PCR products were cleaned up using QIAquick PCR Purification (Qiagen), cloned into pGEM-T easy (Promega, Walldorf, Germany) and subjected to Sanger sequencing using SP6 as reverse primer. 

### 2.7. SNP Array

Genomic DNA was processed on an Infinium^TM^ Global Screening Array-24 v3.0 BeadChip (Illumina, München, Germany) by the Institute of Human Genetics, LIFE&BRAIN GmbH, University of Bonn (Germany). The provided data were called for single nucleotide polymorphisms (SNPs) using GenomeStudio V2.0.5 (Illumina) with a GenCall threshold of 0.2. CNV analysis was performed using GenomeStudio cnvPartition 3.2.0.

### 2.8. Transfection of siRNAs

Gene specific siRNA oligonucleotides were used to modify gene expression levels with reference to AllStars negative Control siRNA (siCTR) obtained from Qiagen. For the knockdown of IRX1 we used Hs_IRX1_3 and Hs_IRX1_7, for IRX3-knockdown we used Hs_IRX3_5 and Hs_IRX3_9, and for KLF15-knockdown Hs_KLF15_6 and Hs_KLF15_9. SiRNAs (80 pmol) were transfected into 1 × 10^6^ cells by electroporation using the EPI-2500 impulse generator (Fischer, Heidelberg, Germany) at 350 V for 10 ms. After 20 h cultivation electroporated cells were harvested for RNA isolation. Another aliquot of the transfected cells was seeded in 96-well plates for proliferation analysis using the IncuCyte v. S3 Live-Cell Analysis System including the software module Cell-By-Cell (Sartorius, Göttingen, Germany). Live-cell imaging experiments were performed twice with four-fold parallel tests. 

## 3. Results

### 3.1. Molecular Subtyping of BC Cell Lines Separate Basal-like from Luminal BC Models

A panel of 29 human BC cell lines originating from primary and metastatic BC (Table 1) was subjected to mRNA-sequencing (RNA-seq) and immunoblotting for expression profiling. Protein expression levels were determined for the well-established biomarkers ER, PR, and HER2 as well as the nuclear hormone receptor AR (androgen receptor) that is discussed as a new clinically relevant biomarker and therapeutic target in BC [48]. Eight of 29 BC cell lines were ER^+^, four of 29 cell lines were PR^+^, 11 of 29 showed strong expression of HER2 (indicated by ++ and +++), another 11 cell lines showed weak expression of HER2 (indicated by +), and eight of 29 cell lines were AR^+^ in the immunoblots (Table 3, Figure S1). From the eight AR^+^ cell lines five were also positive for ER. BT-474, EFM-19 and T-47D presented positive for all tested receptors, whereas 16 cell lines were assigned as TNBC on the basis of their immunoprofiles (Table 3). 

**Figure 1:**
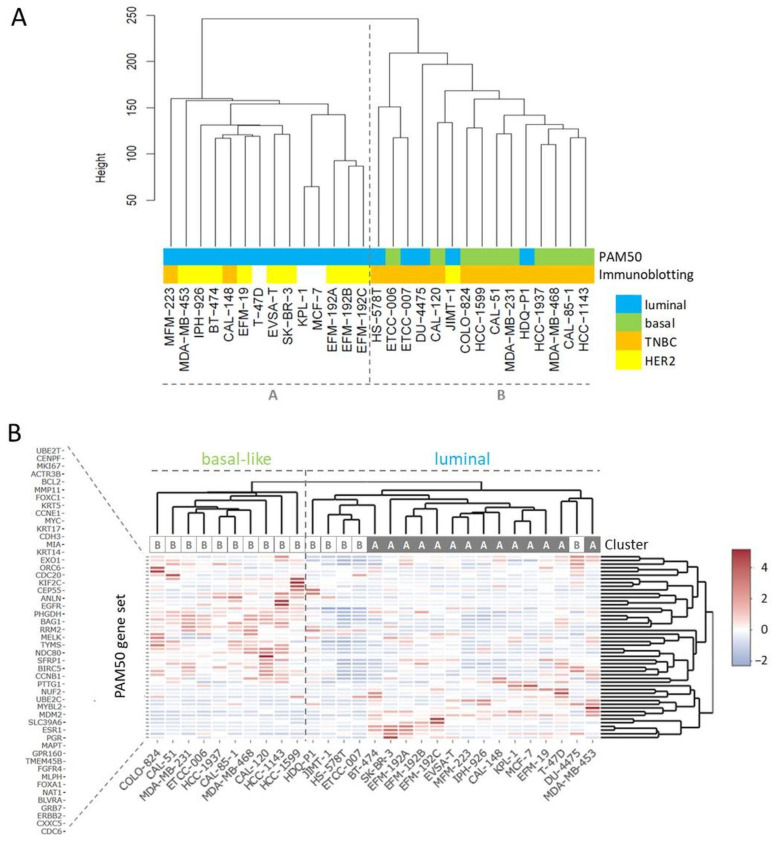
Clustering analyses for subtyping of BC cell lines. (**A**) Transcriptome-wide unsupervised clustering analysis separates BC cell lines into cluster A and cluster B, the assigned PAM50 subtypes are from the analysis shown in (**B**), the immunoblotting data from Table 3. (**B**) Unsupervised clustering analysis using the PAM50 gene set, the indicated cluster is from the transcriptome-wide analysis shown in (**A**).

RNA-seq yielded more than 27 million unique mappable reads per sample. Transcriptome-wide unsupervised clustering analysis revealed that the samples spread in two main branches named cluster A and cluster B (Figure 1A). Cluster A comprised most of the cell lines characterized by strong HER2 expression (indicated by ++ or +++ in Table 3), whereas most of the TNBC cell lines were localized in cluster B (Table 3, Figure 1A). Of note, related cell lines clustered together in the same sub-branches, namely sister cell lines EFM-192A, EFM-192B, and EFM-192C, subclones ETCC-006 and ETCC-007, as well as MCF-7 and its derivative KPL-1. Since the PAM50 gene set is used as a common genetic test for molecular BC subtyping, we applied unsupervised clustering analysis using this set of 50 genes to assign intrinsic molecular subtypes to the BC models (Figure 1B). One of the two main branches was characterized by expression of basal markers like *KRT5* and *KRT17* as well as absence of *ERBB2* (encoding for HER2) expression. Accordingly, the cell lines of this branch were assigned to the basal-like subtype (Figure 1B). 

All cell lines from the basal-like branch were located in cluster B of the transcriptome-wide analysis and were also classified as TNBCs (Figure 1, Table 3). The other main branch from PAM50 clustering analysis contained cell lines expressing typical luminal genes like *ESR1* and *FOXA1* and most cell lines were located in cluster A in the transcriptome-wide cluster dendrogram (Figure 1B). The *ERBB2* strong expressing (>200 tpm) cell lines (BT-474, EFM-192A, EFM-192B, EFM-192C, IPH-926, JIMT-1, MDA-MB-453, SK-BR-3) did not appear as a discrete branch but clustered within the luminal arm. All cell lines positive for AR were located in the luminal arm or cluster A, respectively (Table 3). Of note, individual cell lines, especially JIMT-1 that clustered as HER2 expressing cell line in cluster B, presented mixed molecular phenotypes.

**Figure 2:**
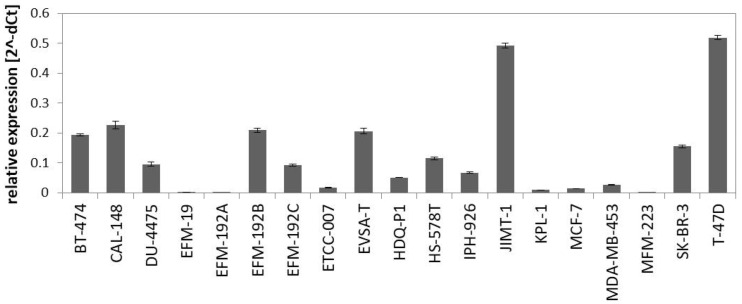
Expression of miR-99a-5p in BC cell lines assigned to luminal subtype in the PAM50 clustering analysis. Expression was determined by qPCR using SNORD48 as endogenous control.

The PAM50 gene set analysis did not allow a sub-classification of the luminal assigned cell lines into LumA and LumB, a phenomenon already observed in previous studies analyzing BC cell lines [9,12]. Nevertheless, we tested a further marker for sub-classification and analyzed expression of miR-99a-5p, which is a predictive tumor suppressor microRNA in BC that was shown to be upregulated in LumA compared to LumB classified BC patients [49,50]. Applying qPCR to determine expression of mature miR-99a-5p in the group of luminal-assigned BC cell lines, we detected the strongest expression of miR-99a-5p in JIMT-1 and T-47D (Figure 2). EFM-19, EFM-192A, ETCC-007, KPL-1, MCF-7, MDA-MB-453, and MFM-223 did not express miR-99a-5p. The observed loss of miR-99a-5p expression might therefore indicate the LumB subtype. However, in contrast to EFM-192A, its sister cell lines EFM-192B and EFM-192C both expressed miR-99a-5p. This indicates that miR-99a-5p expression might substantially vary in different tumor cell clones from the same patient, thus weakening the power of miR-99a-5p to separate LumA from LumB cell lines. Accordingly, intra-tumor heterogeneity could cause the observed differences in miR-99a-5p expression. Furthermore, there is evidence that subtype admixture is relatively common in BC [51]. Therefore, we decided against an assignment of LumA and LumB subtypes to the luminal cell lines based on our transcriptome or miR-99a-5p analyses. 

Thus, transcriptome-wide gene expression analysis of BC cell lines enabled subtyping of single cell lines in two separate clusters. Cluster A was comprised of cell lines with a luminal PAM50 signature, ER^+^ cell lines, and included the cell lines with strong HER2 expression. Cell lines of cluster B often showed a basal-like PAM50 signature and were in all but one case (JIMT-1) TNBCs.

### 3.2. Mutations in BC Cell Lines Frequently Affect TP53 and BRCA2

To characterize the mutational landscape in the panel of BC cell lines, we set up a pipeline for calling SNVs and InDels on RNA-seq data. SNPs with an allele frequency (AF) > 0.01 were filtered as provided by the 1000 genomes project, gnomAD, and dbSNP. Strikingly, amongst mutations at the same site occurring in more than a third of the BC cell lines, many variants showed repetitive elements or low complexity, which might hint at sequencing errors [52,53]. Therefore, mutations present in more than a third of the samples were excluded for further analysis. We also considered the predicted functional effect of a variant and concentrated on variants in coding regions with likely deleterious or damaging consequence for the encoded protein (SIFT ≤ 0.02 or PolyPhen ≥ 0.2).

**Figure 3:**
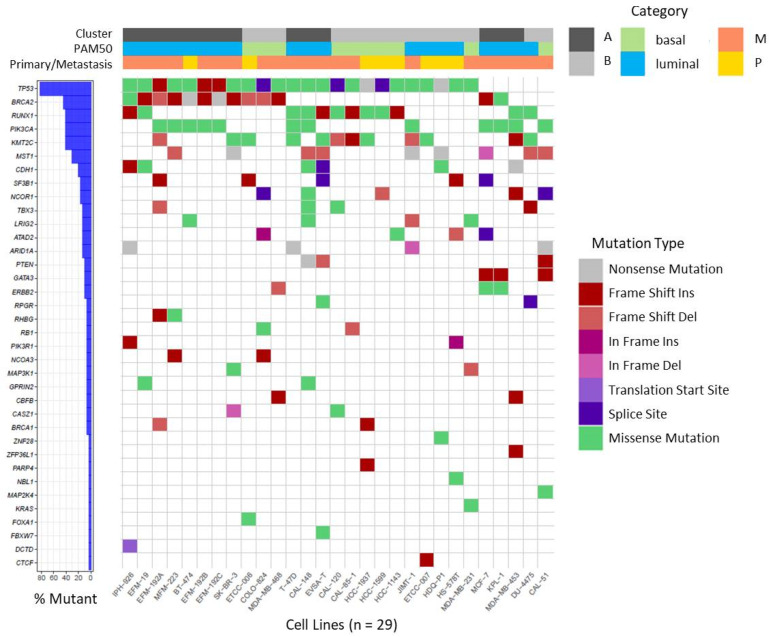
Waterfall plot depicting recurrent non-synonymous mutations in BC cell lines. Mutations were called on RNA-seq data for the gene set reported by Ciriello et al. [54], in *BRCA1* and *BRCA2* and visualized as waterfall plot for the 36 genes in which mutations were identified. For details of identified mutations see Table S2. Assignments of BC cell lines to specific categories (Cluster, PAM50, origin from primary or metastatic tumor) are depicted on top by color codes.

In our BC cell lines we focused on a set of recurrently mutated genes described by Ciriello et al. in primary BC of the TCGA cohort [54] and *BRCA1* and *BRCA2,* as well established BC susceptibility genes [55]. On investigating this set of 70 genes, we found 182 non-synonymous mutations affecting 36 different genes in the 29 BC cell lines (Table S2) which are visualized in Figure 3. The majority of detected variants were missense mutations followed by InDels (Table S2). 

We detected several mutations that were previously described in the respective cell lines, underscoring the plausibility of our variant analysis pipeline. Examples of verified variants are a frame shift insertion in *CDH1* and a missense mutation in *TP53* (c.853G > A) in cell line IPH-926 [27], as well as missense mutations in *PIK3CA* (c.3140A > G) and *TP53* (c.580C > T) in cell line T-47D [56]. Furthermore, several mutations were shared between sister cell lines or sub-clones (Table S2). As observed in BC patients, mutations in the tumor suppressor gene *TP53* are very frequent events in BC [54,57], and were detected in 24 of 29 BC cell lines. Variants in *BRCA2* were also frequently detected in BC cell lines (13 of 29) and mostly affected cell lines assigned to cluster A or luminal subtype, respectively (10 of 13). Mutations in *RUNX1*, *PIK3CA*, and *KMT2C* together ranged at position three affecting 12 of 29 cell lines, each (Figure 3). Mutations in *PIK3CA* were more prevalent in cell lines assigned to luminal BC and were mainly detected in cell lines deriving from metastatic BC. It was also apparent that all *CDH1* mutant cell lines were assigned to luminal BC (6 of 6).

Of note, mutations were called on RNA-seq data, thus allowing the detection of mutations in expressed genes only. Therefore, no conclusive statement on the mutation status of *ACTL6B*, *AQP12A*, *GRIA2*, *HLA-DRB1*, *HRNR*, *IRS4*, *KCNN3*, *OR2D2*, *OR9A2*, and *TCP11* was possible, because the genes were not expressed in most of the cell lines (Figure S2). Nevertheless, mutations were detected even in weakly expressed genes such as *RHBG* and *GPRIN2* (Figure S2). This indicates that for the majority of the 70 genes analyzed, RNA-seq data were indeed suitable to call mutations. 

### 3.3. Identification of Novel Fusion Transcripts in BC Cell Lines

**Figure 4:**
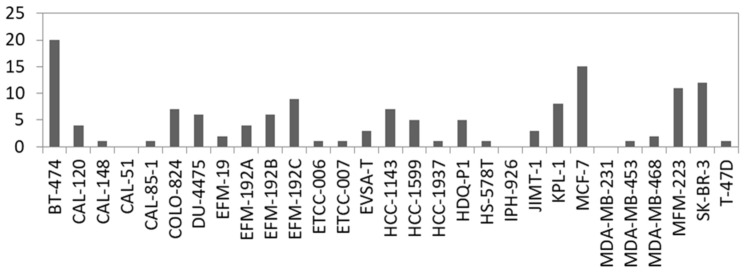
Number of predicted in-frame fusion transcripts per cell line. For details see Table S3.

Fusion genes are the result of genomic structural rearrangements such as translocations or deletions. Recurrent fusion genes were found in BC patients and affect, e.g., *ERBB2* [58]. Therefore, we searched for fusion transcripts applying FusionCatcher on our RNA-seq data based on split reads which are reads that partially align to two distinct locations of the genome. In total, we called 2329 fusions in the 29 BC cell lines which were detected with at least two fusion calling algorithms and with a split read filter > 3. Filtering for in-frame fusion transcripts of relevant expression level (split read filter > 19) resulted in 137 potential fusions ranging from 20 (BT-474) to 0 (CAL-51, IPH-926 and MDA-MB-231) per cell line (Figure 4, Table S3). In concordance with the literature, we detected known fusions in BC cell lines like *ACACA::STAC2* in BT-474, *ARGEF2::SULF2* in MCF-7, and *CYTH1::EIF3H* in SK-BR3 [59]. Consequently, we also called the *ARGEF2::SULF2* fusion in the KPL-1 derivative line of MCF-7 (Table S3). However, not every predicted fusion was shared between derivatives or sister cell lines, which is evident when comparing the absolute number of predicted fusions between, for example, KPL-1 and MCF-7 (Figure 4).

**Figure 5:**
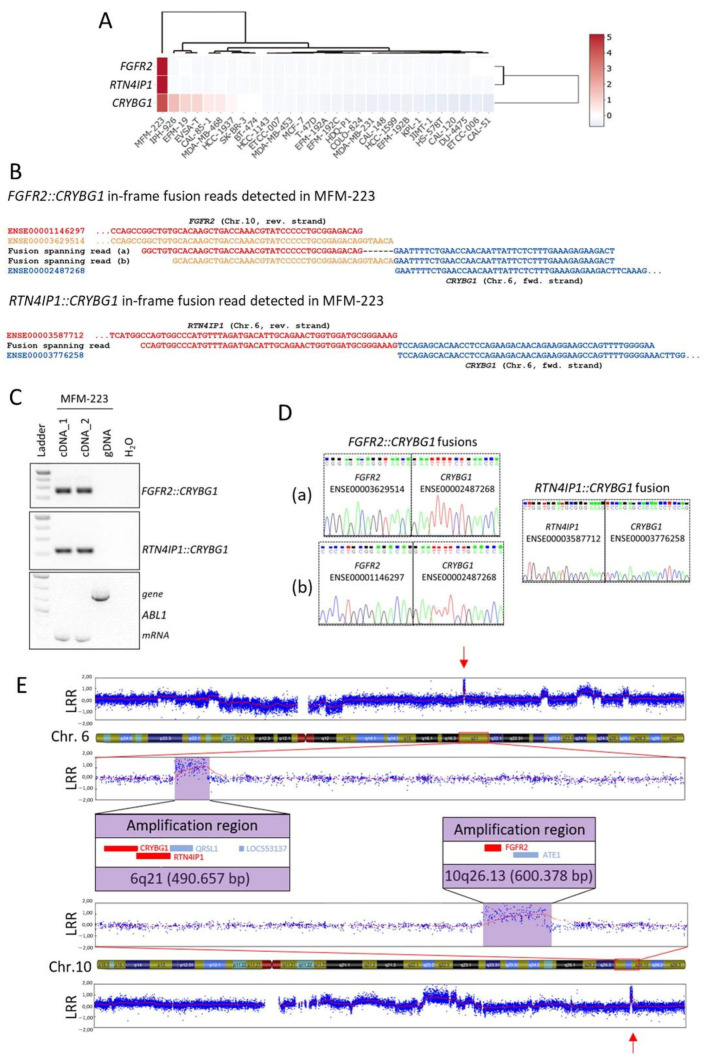
Novel fusion genes involving *CRYBG1* in MFM-223. (**A**) Normalized expression of *CRYBG1*, *FGFR2*, and *RTN4IP1* visualized as heat map in the 29 BC cell lines from RNA-seq analysis. (**B**) Alignment of the detected fusion spanning reads of *FGFR2::CRYBG1* and *RTN4IP1::CRYBG1* to the exons of the fusion partners. (**C**) Fusion gene validation by PCR with primers flanking the fusion spanning read sequences on cDNA prepared from two biological replicates of RNA and on genomic DNA (gDNA) from MFM-223; amplification of *ABL1* served as internal control. (**D**) Electropherogram after cloning and sequencing of PCR products shown in (**C**); (**a**) and (**b**) indicate the two different *FGFR2::CRYBG1* fusions. (**E**) SNP array results showing focal CNAs (red arrows) on Chr.6q21 and Chr.10q26.13 in MFM-223, genes involved in the fusions (*CRYBG1, RTN4IP1, FGFR2*) are depicted in red in the enlarged sections. LRR: Log R ratio.

In order to validate predicted novel fusion transcripts, we selected in-frame fusion transcripts involving *CRYBG1* (Crystallin Beta-Gamma Domain Containing 1, alias *AIM1*) in MFM-223, namely *FGFR2::CRYBG1* and *RTN4IP1::CRYBG1* that were both detected with more than 100 split reads (Table S3). *CRYBG1* has already been identified as a target of genomic aberrations like amplifications and translocations in BC patients [60,61], which further supported the selection of these examples. Expression of *CRYBG1*, *FGFR2* (fibroblast growth factor receptor 2), and *RTN4IP1* (reticulon 4 interacting protein 1) was almost exclusive for MFM-223 across the BC cell lines panel (Figure 5A). 

Due to the usage of alternative splice sites, two different *FGFR2::CRYBG1* in-frame fusion transcripts were called that differed only by six nucleotides in length of the *FGFR2* sequence (exons ENSE00001146297 and ENSE00003629514, respectively) fused to *CRYBG1* exon ENSE00002487268 (Figure 5B). The intra-cellular kinase domain of FGFR2 is encoded downstream of the affected *FGFR2* exons, therefore both *FGFR2::CRYBG1* transcripts lack the kinase domain. The other detected fusion spanning read called exon ENSE00003587712 of *RTN4IP1* fused to ENSE00003776258 of *CRYBG1* (Figure 5B). With primers flanking the fusion spanning read sequences in the respective complementary exons, the presence of the fusion transcripts was validated on cDNA from MFM-223 (Figure 5C). No PCR product was obtained from gDNA of MFM-223 which indicates that the breakpoints are located in the flanking introns of the fusion partners. Sanger sequencing of cloned PCR products confirmed the existence of the two different in-frame *FGFR2::CRYBG1* fusions and the in-frame *RTN4IP1::CRYBG1* fusion (Figure 5D). Interestingly, *CRYBG1* and *RTN4IP1* are neighboring genes on Chr.6q21 that are encoded on the forward and reverse strand, respectively, indicating that several breakpoints in this region were required to form the detected fusion. Furthermore, amplifications of *CRYBG1* were reported for TNBC patients [60]. Thus, to test for copy number aberrations (CNAs) of the genomic regions involved in the identified fusion transcripts, we performed SNP array analysis from gDNA of MFM-223 (call rate > 97% Figure S3). Interestingly, the analysis indicated focal chromosomal amplifications of both loci which encode for the genes involved in the identified fusion transcripts with a region spanning ~491 kB on 6q21 and ~600 kB on 10q26.13, respectively (Figure 5E). The amplifications might also be a reason for the strong expression of *CRYBG1*, *FGFR2*, and *RTN4IP1* identified in MFM-223 (Figure 5A). Importantly, loci of focal CNAs are frequently enriched for cancer driver genes [62], indicating a potential oncogenic role of the identified loci in MFM-223. 

In sum, the prediction of fusion transcripts from RNA-seq data enabled us to identify novel fusion genes involving *CRYBG1* in the TNBC cell line MFM-223 which might have an oncogenic potential due to their location in focal amplified regions and their strong expression. 

### 3.4. Identification of IRX Genes and KLF15 as Candidate Tumor Suppressor Genes in BC

Homeobox genes including Iroquois homeobox (*IRX*) genes encode transcription factors which are involved in normal developmental processes but also in cancerogenesis when deregulated. Recently, we showed conspicuous expression of *IRX1* in the pre-B-cell stage of lymphopoiesis and revealed aberrant expression of *IRX1*, *IRX2,* and *IRX3* in patients and cell lines of pre-B-cell leukemia [63]. Furthermore, we identified the Krüppel-like Zinc-finger factor KLF15 as an aberrant transcriptional activator of *IRX3* in these leukemic cells [63,64,65,66].

**Figure 6:**
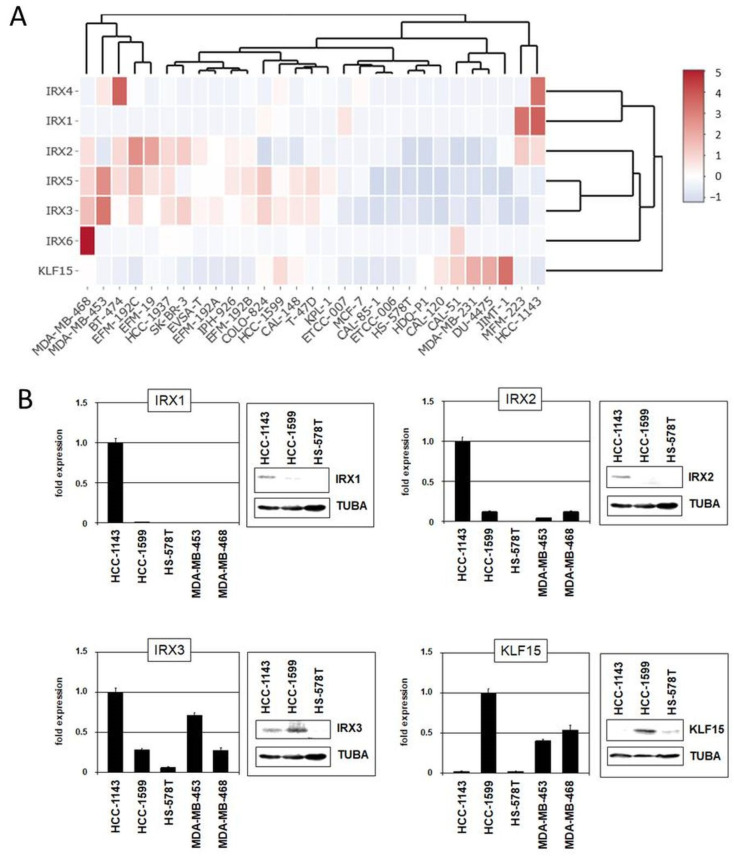
Gene expression pattern of *IRX* genes and *KLF15* in BC cell lines. (**A**) Heatmap showing gene expression levels according to our RNA-seq data. The genes and cell lines are clustered. (**B**) Expression analyses by qPCR and immunoblot in selected BC cell lines for IRX1, IRX2, IRX3, and KLF15.

The family of *IRX* homeobox genes consists of six members, *IRX1–IRX6*, representing developmental regulators in particular tissues and stages. Here, we analyzed the expression of all six *IRX* genes in addition to *KLF15* in normal breast tissue (Figure S4), as well as in BC patients and cell lines using published gene expression and our RNA-seq data (Figure 6A and Figures S5 and S6). These data demonstrated physiological activity of *KLF15* and all *IRX* genes in breast tissue while subsets of BC patients and cell lines showed aberrant downregulation, suggesting that these genes may represent candidate tumor suppressors. Expression analysis by qPCR and immunoblotting of selected BC cell lines confirmed downregulation of *IRX1*, *IRX2*, *IRX3,* and *KLF15* in some samples (Figure 6B). 

**Figure 7:**
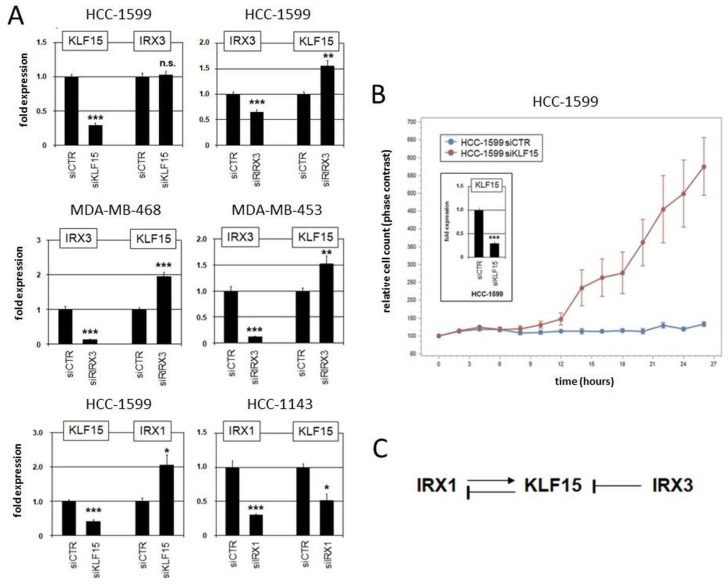
Regulatory role of *IRX1*, *IRX3*, and *KLF15* in BC cell lines. (**A**) Gene expression analysis by qPCR in HCC-1599, HCC-1143, MDA-MB-468, and MDA-MB-453 treated for siRNA-mediated knockdown of *KLF15*, *IRX1*, and *IRX3*. (**B**) Proliferation analysis by live-cell imaging in HCC-1599 treated for siRNA-mediated knockdown of *KLF15* (confirmed by qPCR analysis; insert), indicating an activating impact on cell proliferation. Note that untransfected HCC-1599 cells have a doubling time of 4 to 5 d. (**C**) Schematic diagram summarizing the regulatory relationships of *IRX1*, *KLF1*, and *IRX3* observed in BC cell lines. * *p* < 0.05, ** *p* < 0.01, *** *p* < 0.001, n.s. not significant.

Moreover, we performed siRNA-mediated knockdown of *KLF15*, *IRX1,* and *IRX3* in suitable cell lines to investigate their regulatory relationship (Figure 7). The results indicated that *KLF15* failed to regulate *IRX3* in HCC-1599 but mediated inhibition of *IRX1* in HCC-1143. On the other hand, *IRX1* mediated activation while *IRX3* was involved in repression of *KLF15* (Figure 7A). Repression of *KLF15* by IRX3 was also detected in MDA-MB-468 and MDA-MB-453 (Figure 7A). Thus, we revealed specific regulatory connections between these candidate tumor suppressor genes in BC which differ from their activity reported in pre-B-cell leukemia [63]. Finally, proliferation analysis by live-cell imaging of BC cell line HCC-1599 treated for knockdown of *KLF15* demonstrated a repressive role of this transcription factor in proliferation (Figure 7B), supporting its tumor suppressor status in BC. Our observed regulatory relationships in BC cell lines are summarized in a diagram (Figure 7C).

## 4. Discussion

Continuous cell lines play an important role in BC research. Due to the heterogeneity of BC, knowledge about the molecular characteristics of BC cell lines is essential for selection of a suitable in vitro model. This study investigated the molecular landscape of a panel of 29 authenticated and publicly available BC cell lines that can now easily be considered during cell line selection, especially as we made processed gene expression data accessible via the open webtool DSMZCellDive. 

BC comprises different molecular subtypes. As reported in several previous studies, BC cell lines share many of the molecular characteristics of primary BC including the subtypes [9]. Accordingly, we could successfully assign molecular subtypes to our panel of BC cell lines applying transcriptome-wide gene expression analysis in combination with immunoprofiling for ER, PR, AR, and HER2. Interestingly, a substantial number (38%) of the analyzed BC cell lines showed weak expression of HER2, which therefore might serve as models for the recently discussed group of HER2-low TNBC patients that were shown to benefit from novel therapies with antibody–drug conjugates (Trastuzumab deruxtecan) [67]. In our study on cell lines the power of transcriptome-wide unsupervised hierarchical clustering outperformed the PAM50 clustering analysis in respect to the assignment to the main molecular subtypes. This is not surprising as the PAM50 gene set contains genes like *MKI67*, whose expression in cell lines is less informative because transcript levels do not vary as much as in tissue samples. In line with reports from others [12], discrimination between LumA and LumB subtypes in cell lines was not possible. Of note, typical LumA tumors are not well represented by BC cell lines because they usually do not grow in vitro [24]. Therefore, the differences that can be observed in PAM50 sub-branches between primary BC samples and BC cell lines can partially be attributed to the lack of characteristic LumA cell lines. However, individual cell lines (e.g., JIMT-1) demonstrated mixed molecular phenotypes, a fact that should be considered in the selection process of cell lines as models. 

Using our RNA-seq data we investigated the mutation status of a set of 70 genes that was previously shown to be implicated in BC. We frequently detected mutations affecting *TP53* and *BRCA2* in the studied BC cell lines panel. Both genes encode for tumor suppressor proteins involved in DNA repair that are known BC susceptibility genes [55]. Studies in primary BC showed that *TP53* mutations were most frequently found in basal-like (80%) and HER2-enriched (72%) tumors [57]. Accordingly, all except one basal-like BC cell line from our panel were mutated in *TP53*. However, also many cell lines of luminal subtype, including the HER2 positive cell lines, harbored mutations in *TP53* indicating that mutations in *TP53* are a rather common feature of BC cell lines. Incorvaia et al. reported in a cohort of 531 BC patients that pathogenic variants of *BRCA2* were often found in tumors assigned to luminal BC, especially LumB subtype [68]. We found primarily *BRCA2* mutations in cell lines assigned to luminal BC, thus indeed reflecting the situation observed in primary tumors. *RUNX1*, *PIK3CA,* and *KMT2C* were also frequently affected by mutations in the panel of BC cell lines. In patients, mutations in *RUNX1* were more prevalent in luminal and HER2-enriched tumors and absent in basal-like BC [57]. In contrast, in the analyzed cell lines, *RUNX1* mutations were present in both, luminal and basal-like models. However, in agreement with previous studies in BC patients [54,57], we found most *PIK3CA* mutations in cell lines assigned to luminal subtype. Also, the *CDH1*-mutant cell lines identified were assigned to luminal subtype. In patients with LumA subtype, *CDH1* belongs together with *PIK3CA*, *MAP3K1*, *GATA3*, *TP53,* and *MAP2K4* to the most frequently mutated genes [57]. In summary, our mutation analyses confirmed that the cell lines share relevant characteristics with primary BC and are thus suitable in vitro models although not all heterogeneity of BC can be reflected. 

We identified two novel fusion transcripts involving *CRYBG1* in the cell line MFM-223. *CRYBG1* (alias *AIM1*, absent in melanoma), located on Chr.6q21, was initially identified as a frequent target of LOH and tumor suppressor in melanoma [69,70]. In contrast, *CRYBG1* was found as a target of genomic aberrations like amplifications and translocations in BC patients. Lips et al. reported amplifications of *CRYBG1* in three of 50 patients with TNBC [60]. Recently, a novel unique *FGFR2::CRYBG1* fusion was detected in a BC patient [61]. FGFR2 belongs to the FGFR family of receptor tyrosine kinases of which members were found to be fused to a variety of translocation partners in multiple cancers [71]. We report here that the TNBC cell line MFM-223 carries an in-frame *FGFR2::CRYBG1* fusion, making it a good model to study the role of *FGFR2::CRYBG1* fusions. Interestingly, the *FGFR2::CRYBG1* fusion in MFM-223 lacks the tyrosine kinase domain of FGFR2 but fuses the extracellular and transmembrane part of FGFR2 to the C-terminal domain of CRYBG1. CRYBG1 interacts with the cytoskeleton and its C-terminal domain is required for the binding to β-actin [72,73]. In prostate epithelial cells, CRYBG1 is strongly associated with the actin cytoskeleton and its depletion affects cytoskeletal remodeling, migration, invasion, and anchorage-independent growth [72]. 

The second fusion partner of *CRYBG1* identified in this study in MFM-223 was *RTN4IP1* (alias NIMP). RTN4IP1 is a mitochondrial NADPH oxidoreductase and mutations in *RTN4IP1* decrease mitochondrial respiratory complex I and IV activities [74,75,76]. It was recently shown that *RTN4IP1* is over-expressed in BC tissue and that high expression levels of *RTN4IP1* predict an adverse prognosis in BC [77]. In the analyzed BC cell lines panel *RTN4IP1* only showed increased expression in MFM-223. *CRYBG1* and *RTN4IP1* are neighboring genes on Chr.6q21 which are encoded on the forward and reverse strand, respectively. Therefore, the identified in-frame *CRYBG1::RTN4IP1* fusion transcript is an indicator of a more complex chromosomal alteration involving 6q21. 

Importantly, *CRYBG1* and its two identified fusion partners were located in regions of focal CNA on 6q21 and 10q26.13 in MFM-223. Focal CNAs often harbor cancer driver genes [62], supporting a possible oncogenic role of the identified *CRYBG1* fusion transcripts. Furthermore, fusion transcripts involving oncogenes have been shown to exhibit increased expression in cancer [58], and we observed indeed strong expression of *CRYBG1*, *FGFR2,* and *RTN4IP1* in MFM-223. The oncogene hypothesis is further supported by CRISPR loss-of-function screens from DepMap (https://depmap.org/), in which *CRYBG1* and *FGFR2* are among the top 10 preferentially essential genes for cell growth of MFM-223.

Our comprehensive expression analyses indicated that *KLF15* and all *IRX* genes are candidate tumor suppressors in BC. The data confirmed previous studies which analyzed selected IRX members and the proliferative role of KLF15 [78,79,80,81]. Moreover, our data show that particular *IRX* genes and *KLF15* operate in a breast (cancer) specific regulatory network. Therefore, these genes may represent diagnostic markers for BC subsets or stages, however, deserving additional evaluation. BC cell lines recapitulated the situation observed in patients serving as suitable models for functional studies.

Despite the above-mentioned novel knowledge gained from the data presented, it should be noted that the panel of BC cell lines studied does not cover well the histological and molecular heterogeneity of BC. For example, only IPH-926 was derived from breast lobular carcinoma. All other BC cell lines represented ductal carcinoma. With the exception of ETCC-006 and ETCC-007, which were established from the same patient diagnosed with DCIS using hTERT, all other cell lines in the panel were derived from invasive BC. Furthermore, the majority (72%) of BC cell lines in the panel were established from metastatic late stage tumors. In principle, however, these limitations are more a symptom of the fact that still most BC cell lines have been successfully established from advanced-stage tumors and pleural effusions [9]. To cover the heterogeneity of BC we need more BC cell lines, especially from less advanced, untreated BC from diverse populations. It is not limited to the field of BC research that many more and also well characterized cancer cell lines are needed for in vitro research, especially in respect to the development of targeted therapies [82]. Furthermore, the tumor microenvironment has a strong influence on growth and survival of tumor cells. Therefore, the artificial culture conditions may effect molecular differences observed between cell lines and primary BC [12]. In summary, this points out that it is even more relevant to consider the cell line characteristics presented here and elsewhere, in order to select a proper cell line model for a specific research question. Most research is still conducted with a very few (old) BC cell lines like MCF-7 [83], presumably because more molecular data are available from the literature for the commonly used models. We therefore believe that the data provided in this study will expand the selection of suitable models. 

## 5. Conclusions

Our study depicts the molecular landscapes, consisting of gene expression profiles, mutation patterns, and potential in-frame fusion genes for a panel of 29 publicly available BC cell lines. With the application to selected examples we demonstrated the usefulness of the data to gain novel insights into cancer relevant genes. In general, the determined molecular characteristics might serve as a valuable decision aid to improve the selection of appropriate models for BC research.

## Figures and Tables

**Table 1:** BC cell lines panel.

Cell Line	Origin	Stage	Histologic Subtype	Sex	Age	Site of Sampling	P/M	Refs
BT-474	breast ductal carcinoma	invasive	ductal	f	60	breast	P	[17]
CAL-120	breast adenocarcinoma	invasive	na	f	43	PE	M	*
CAL-148	breast adenocarcinoma	invasive	ductal	f	58	PE	M	[18]
CAL-51	breast adenocarcinoma	invasive	ductal	f	45	PE	M	[19]
CAL-85-1	breast adenocarcinoma	invasive	ductal	f	35	breast	M	[18]
COLO-824	breast carcinoma	invasive	na	f	52	PE	M	*
DU-4475	breast ductal carcinoma	invasive	ductal	f	62	skin	M	[20]
EFM-19	breast ductal carcinoma	invasive	ductal	f	50	PE	M	[21]
EFM-192A ^1^	breast adenocarcinoma	invasive	na	f	46	PE	M	*
EFM-192B ^1^	breast adenocarcinoma	invasive	na	f	46	PE	M	*
EFM-192C ^1^	breast adenocarcinoma	invasive	na	f	46	PE	M	*
ETCC-006 ^2^	breast ductal carcinoma	in situ	ductal	f	47	breast	P	[22]
ETCC-007 ^2^	breast ductal carcinoma	in situ	ductal	f	47	breast	P	[22]
EVSA-T	breast carcinoma	invasive	na	f	58	ascites	M	[23]
HCC-1143 ^3^	breast ductal carcinoma	invasive	ductal	f	52	breast	P	[24]
HCC-1599 ^3^	breast ductal carcinoma	invasive	ductal	f	44	breast	P	[24]
HCC-1937 ^3^	breast ductal carcinoma	invasive	ductal	f	24	breast	P	[24]
HDQ-P1	breast ductal carcinoma	invasive	ductal	f	50	breast	P	[25]
HS-578T	breast carcinosarcoma	invasive	ductal	f	74	breast	P	[26]
IPH-926	breast lobular carcinoma	invasive	lobular	f	72	ascites	M	[27]
JIMT-1	breast ductal carcinoma	invasive	ductal	f	62	PE	M	[28]
KPL-1 ^4^	breast adenocarcinoma	invasive	na	f	69	PE	M	[10]
MCF-7	breast adenocarcinoma	invasive	na	f	69	PE	M	[10]
MDA-MB-231	breast carcinoma	invasive	na	f	51	PE	M	[29]
MDA-MB-453	breast carcinoma	invasive	na	f	48	PF	M	[29]
MDA-MB-468	breast carcinoma	invasive	na	f	51	PE	M	[29]
MFM-223	breast ductal carcinoma	invasive	ductal	f	>45	PE	M	[30]
SK-BR-3	breast adenocarcinoma	invasive	na	f	43	PE	M	[31]
T-47D	breast ductal carcinoma	invasive	ductal	f	54	PE	M	[32]

^1^ Sister cell lines from same patient (A was established 14 days earlier than B and C). ^2^ hTERT immortalized clones from same patient. ^3^ Paired B lymphoblastoid cell line (B-LCL) available. ^4^ Derivative of MCF-7 (see DSMZ website for details). * Data provided by cell line depositor to DSMZ cell lines bank. Abbreviations: f = female, M = metastatic tumor, na = not available, P = primary tumor, PE = pleural effusion, PF = pericardial fluid.

**Table 2:** Primers used for fusion gene analysis.

Name	Sequence (5′→3′)	Product Size [bp]
FGFR2_ex9_fwd ^1^	TGTATGGTGGTAACAGTCATCC	240 and 246
CRYBG1_ex18_rev ^2^	CTGAACAGAGCGTATTTGTGTG	
RTN4IP1_ex8_fwd ^3^	GGAAAGGAGTCCATTATCGCTG	206
CRYBG1_ex3_rev ^2^	TGATCTGGTGGGACTCTCTAAC	
ABL1_fwd	TGACTTTGAGCCTCAGGGTCTGAGTGAAGCC	216 (mRNA) 779 (gene)
ABL1_rev	CCATTTTTGGTTTGGGCTTCACACCATTCC	

^1^ Exon number refers to ENST00000683211.1. ^2^ Exon number refers to ENST00000633556.3. ^3^ Exon number refers to ENST00000369063.8.

**Table 3:** Determined molecular characteristics of BC cell lines. Signal intensity indicated by +, ++, and +++ for ER, PR, AR, and HER2 protein as determined by densitometric analysis of immunoblots and in relation to signals of BT-474 (Figure S1). TNBC status is defined by absence of ER and PR and a weak (+) or absent HER2 signal. Assignment of cell lines to cluster and PAM50 subtype is based on unsupervised clustering analysis presented in Figure 1.

Cell Line	ER	PR	AR	HER2	TNBC	Cluster	PAM50 Subtype
BT-474	++	++	++	+++		A	luminal
CAL-120					yes	B	basal-like
CAL-148			+	+	yes	A	luminal
CAL-51				+	yes	B	basal-like
CAL-85-1				+	yes	B	basal-like
COLO-824				+	yes	B	basal-like
DU-4475					yes	B	luminal
EFM-19	+++	++	++	++		A	luminal
EFM-192A	+			+++		A	luminal
EFM-192B	+			+++		A	luminal
EFM-192C	++	+	+	+++		A	luminal
ETCC-006					yes	B	basal-like
ETCC-007				+	yes	B	luminal
EVSA-T				++		A	luminal
HCC-1143				+	yes	B	basal-like
HCC-1599				+	yes	B	basal-like
HCC-1937				+	yes	B	basal-like
HDQ-P1				+	yes	B	luminal
HS-578T					yes	B	luminal
IPH-926				++		A	luminal
JIMT-1				++		B	luminal
KPL-1	+++		+++	+		A	luminal
MCF-7	+++			+		A	luminal
MDA-MB-231					yes	B	basal-like
MDA-MB-453			+++	++		A	luminal
MDA-MB-468					yes	B	basal-like
MFM-223			+++		yes	A	luminal
SK-BR-3				++		A	luminal
T-47D	+++	+++	++	++		A	luminal

## Data Availability

Data generated for this study were made publicly available. Raw RNA-seq data were deposited at BioStudies under S-BSST1200C and processed expression data are available at DSMZCellDive (https://celldive.dsmz.de/). Bioinformatic pipelines and data are available from the corresponding author upon request.

## Supplementary Materials

The following supporting information can be downloaded at: https://www.mdpi.com/article/10.3390/cells13040301/s1, Table S1: Cell culture media and cultivation conditions; Figure S1: Immunoblot analyses of AR, ER, HER2, and PR in 29 BC cell lines; Table S2: Details for the mutations depicted in the waterfall plot identified in the 29 BC cell lines; Figure S2: Expression heat map for the gene set analyzed for mutations; Table S3: Predicted in-frame fusion transcripts in BC cell lines; Figure S3: SNP array data for MFM-223; Figure S4: RNA-seq data for six IRX genes and KLF15 from different normal tissues; Figure S5: Gene expression profiling data for normal breast tissue, primary BC samples and BC cell lines from GSE45827; Figure S6: Gene expression profiling data for normal breast tissue, primary BC samples and BC cell lines from GSE65216.
