# Supplementary material for: Molecular Characterization and Subtyping of Breast Cancer Cell Lines Provide Novel Insights into Cancer Relevant Genes
Source: Cells. 2024 Feb 6;13(4):301. doi: 10.3390/cells13040301 (PMC10886524; doi:10.3390/cells13040301)
Supplement: Supplementary file 1 [file cells-13-00301-s001.zip › Supl_Figures_final.pdf]

Figure S1

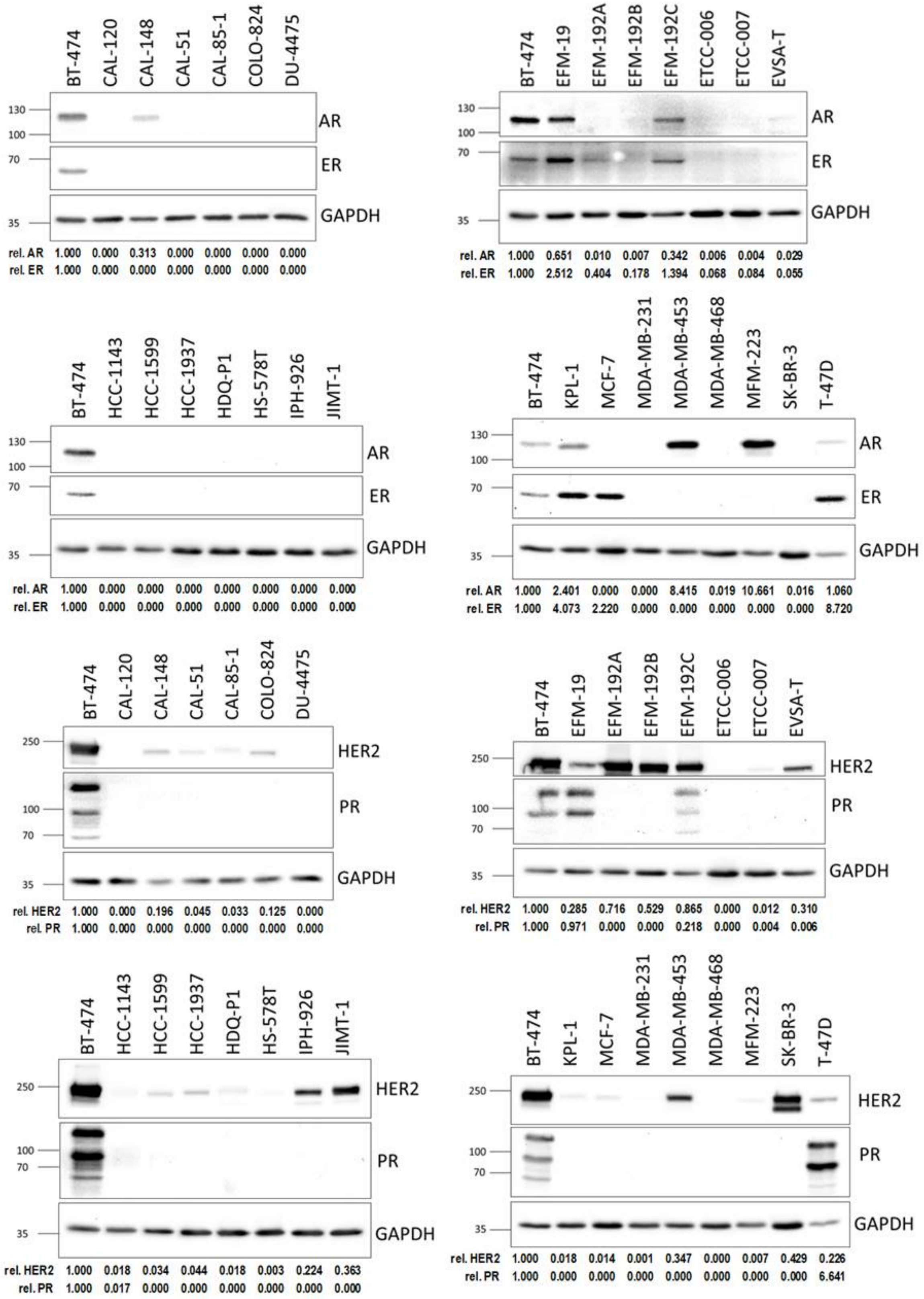

Figure S1. Immunoblot analyses of AR, ER, HER2 and PR in 29 BC cell lines. Cell line BT-474 is positive for all analyzed proteins and was therefore loaded on each Western blot as positive control and to enable a comparison between the different blots. Signal intensities in all cell lines were assessed in relation to BT-474 protein levels which were set to 1. Numbers below the immunoblots refer to the relative signal intensities as determined by densitometric analysis using ImageJ, while GAPDH levels served as loading controls. Numbers on the left refer to the corresponding protein marker bands in kDa.

Figure S2

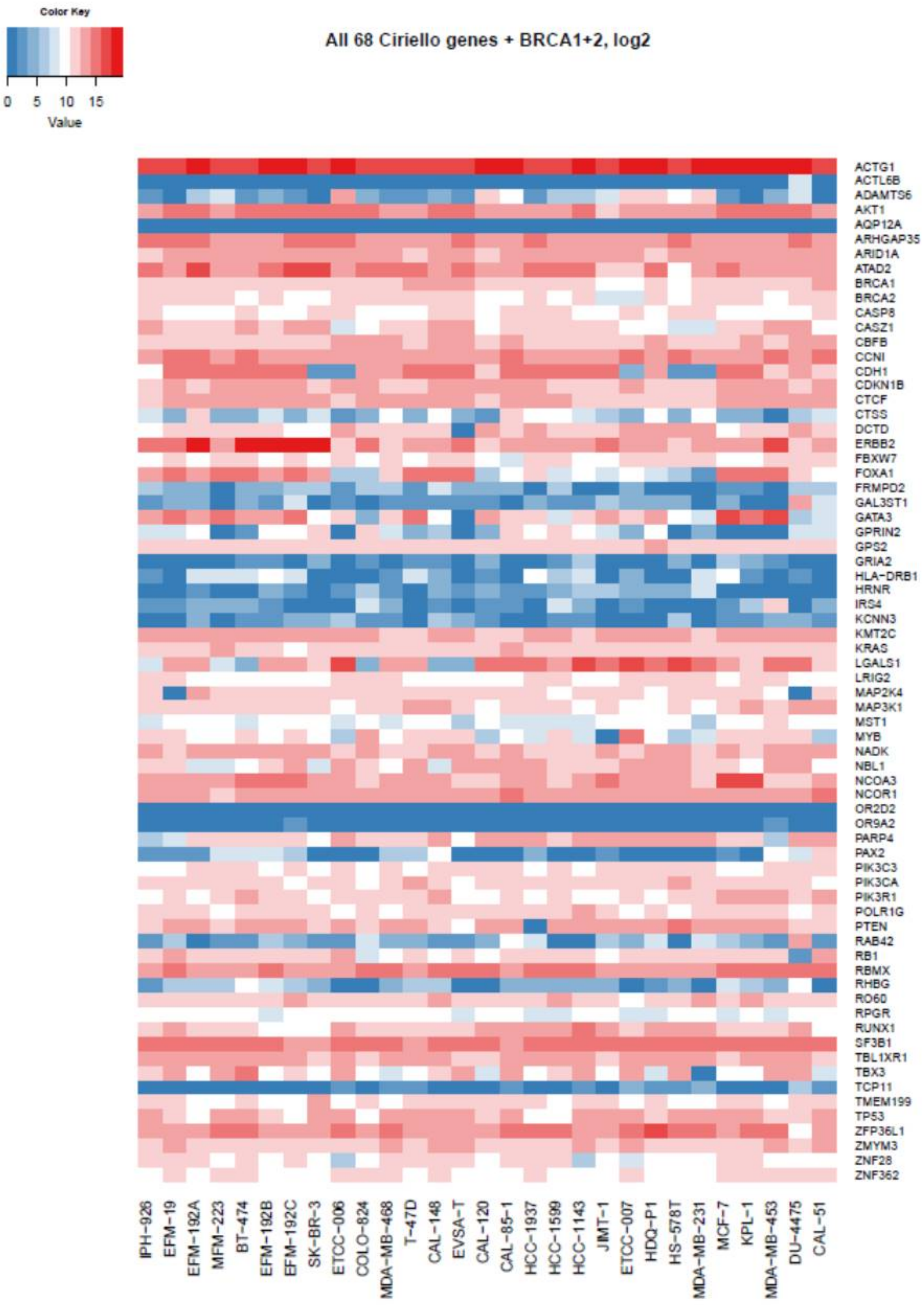

Figure S2. Expression heat map for the gene set analyzed for mutations. Normalized RNA-seq data were log2 transformed after adding 1 for avoiding non-defined values. Genes are ordered alphabetically and cell lines are ordered analogously as in Figure 3.

Figure S3

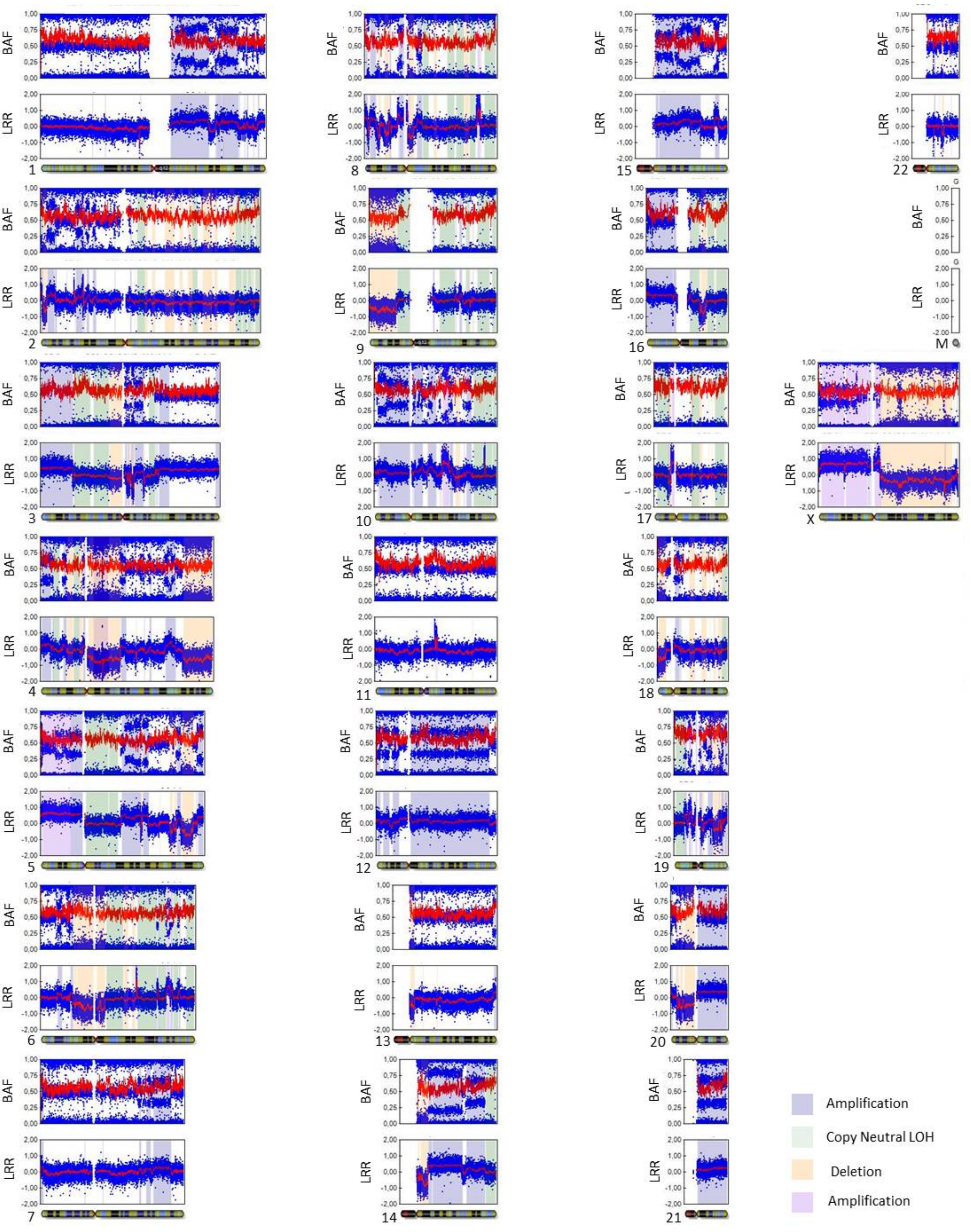

Figure S3. SNP array data for MFM-223. Overview of CNV analysis depicting B allele frequency (BAF) and LogR ratio (LRR) for each chromosome. Shaded regions in different colors indicate: blue: Amplification (CNV value of 3), green: Copy Neutral LOH (CNV value of 2), yellow: Deletion (CNV value of 1), purple: Amplification with higher intensity compared to the mean value (CNV value of 4). The results include copy number events, even when they are smaller than 350.000 bp.

Figure S4

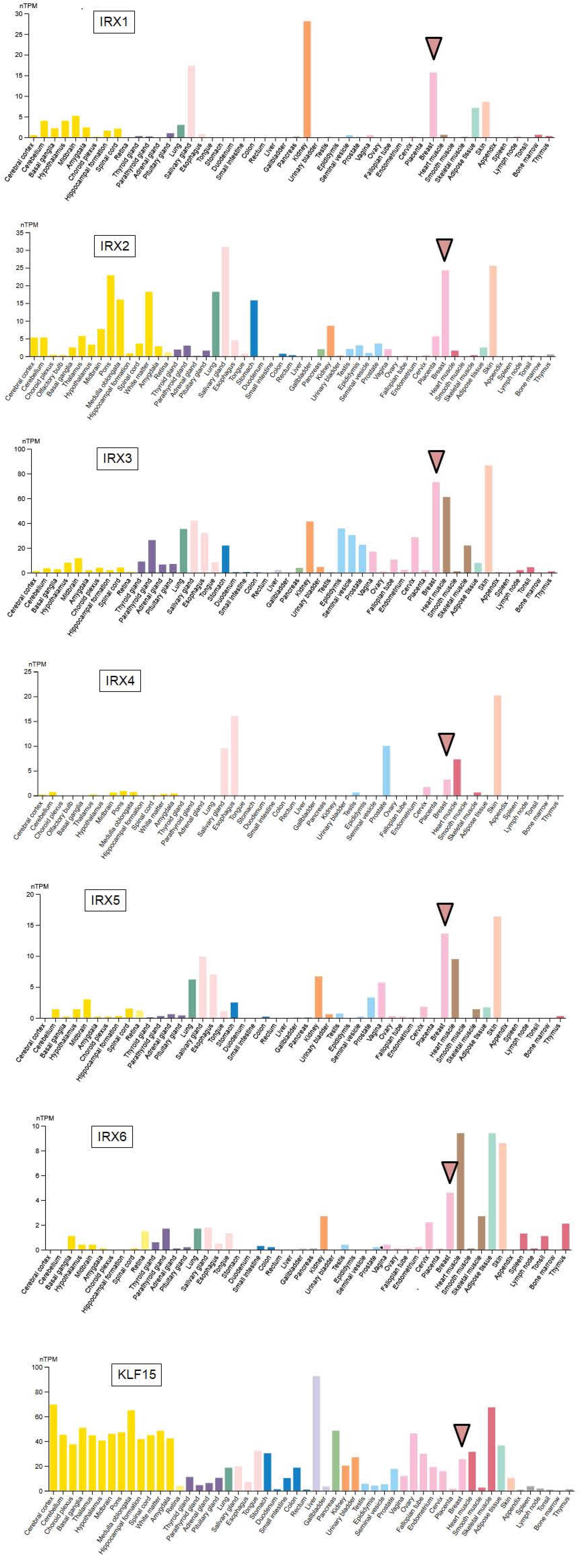

Figure S4: RNA-seq data for six IRX genes and KLF15 from different normal tissues. The data were obtained from the Human Protein Atlas. Breast tissue is indicated by arrow heads.

GEO: expression profiling (GSE45827)

Figure S5

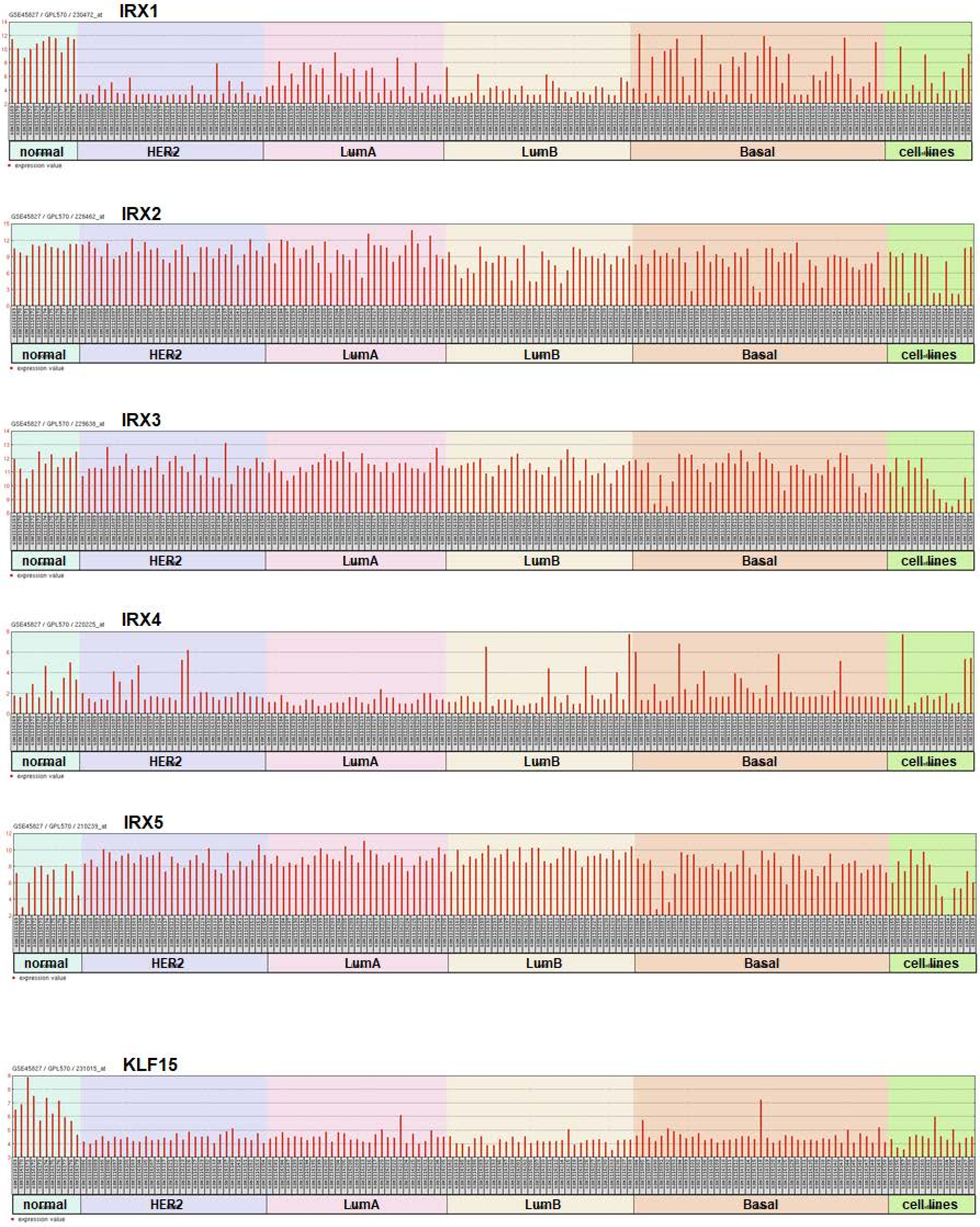

Figure S5: Gene expression profiling data (obtained from GEO data set GSE45827) for normal breast tissue and breast cancer samples, classified as HER2-positive (HER2), Luminal A (LumA), Luminal B (LumB), Triple negative (TNBC), and breast cancer cell lines (cell lines).

GEO: expression profiling (GSE65216)

Figure S6

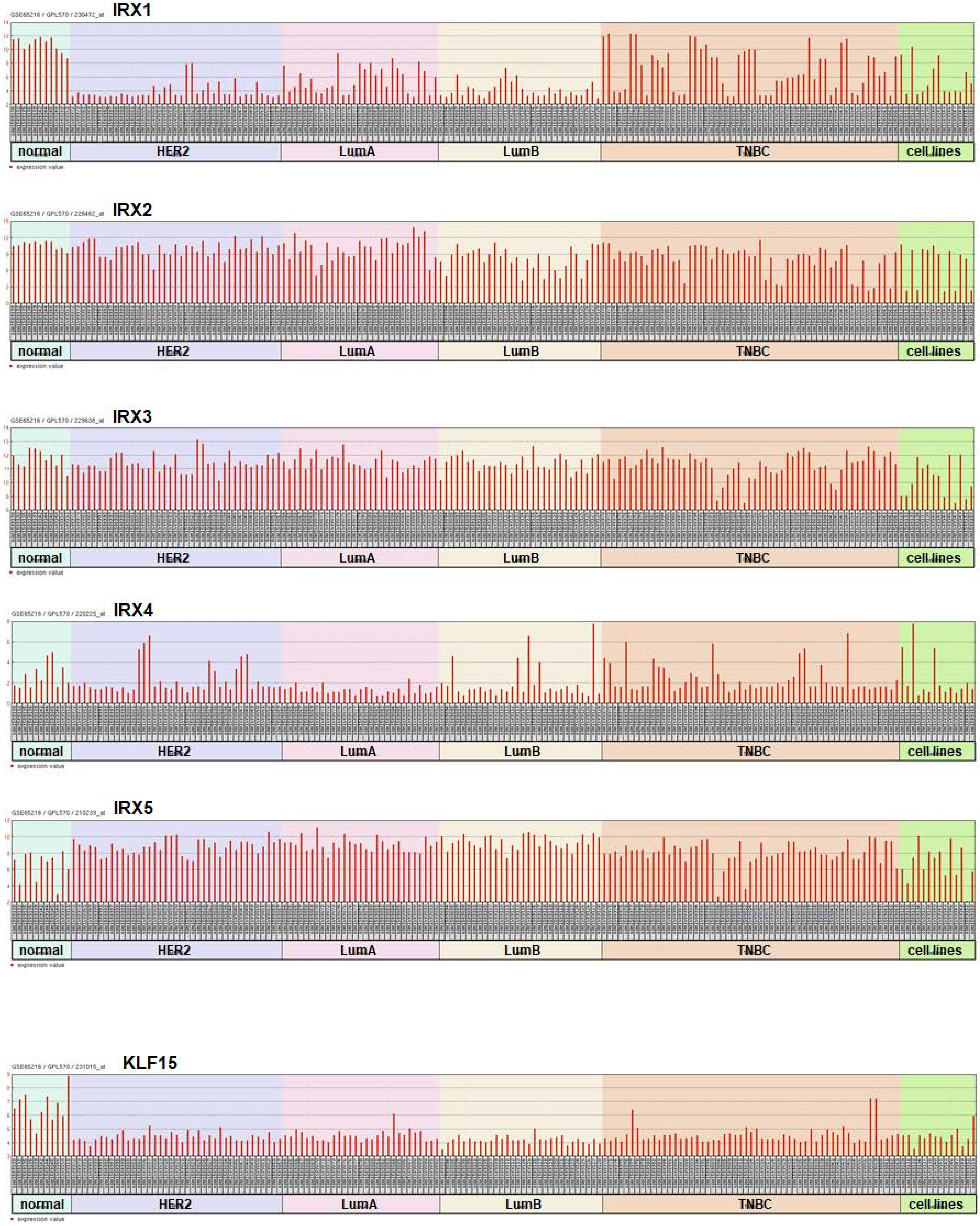

Figure S6: Gene expression profiling data (obtained from GEO data set GSE65216) for normal breast tissue and breast cancer samples, classified as HER2-positive (HER2), Luminal A (LumA), Luminal B (LumB), Triple negative (TNBC), and breast cancer cell lines (cell lines).
